# Gut Microbiome and Bile Acid Metabolism Induced the Activation of CXCR5+ CD4+ T Follicular Helper Cells to Participate in Neuromyelitis Optica Spectrum Disorder Recurrence

**DOI:** 10.3389/fimmu.2022.827865

**Published:** 2022-01-20

**Authors:** Xi Cheng, Luyao Zhou, Zhibin Li, Shishi Shen, Yipeng Zhao, Chunxin Liu, Xiaonan Zhong, Yanyu Chang, Allan G. Kermode, Wei Qiu

**Affiliations:** ^1^ Department of Neurology, Third Affiliated Hospital of Sun Yat-sen University, Guangzhou, China; ^2^ Centre for Neuromuscular and Neurological Disorders, Perron Institute, The University of Western Australia, Perth, WA, Australia; ^3^ Institute for Immunology and Infectious Diseases, Murdoch University, Murdoch, WA, Australia

**Keywords:** T follicular helper cells, neuromyelitis optica spectrum disorder, CXCL13, bile acid, gut microbiota

## Abstract

From the perspective of the role of T follicular helper (Tfh) cells in the destruction of tolerance in disease progression, more attention has been paid to their role in autoimmunity. To address the role of Tfh cells in neuromyelitis optica spectrum disorder (NMOSD) recurrence, serum C-X-C motif ligand 13 (CXCL13) levels reflect the effects of the Tfh cells on B-cell-mediated humoral immunity. We evaluated the immunobiology of the CXCR5+CD4+ Tfh cells in 46 patients with NMOSD, including 37 patients with NMOSD with an annual recurrence rate (ARR) of<1 and 9 patients with NMOSD with an ARR of ≥1. Herein, we reported several key observations. First, there was a lower frequency of circulating Tfh cells in patients with an ARR of<1 than in those with an ARR of ≥1 (P< 0.05). Second, the serum CXCL13 levels were downregulated in individuals with an ARR<1 (P< 0.05), processing the ability to promote Tfh maturation and chemotaxis. Third, the level of the primary bile acid, glycoursodeoxycholic acid (GUDCA), was higher in patients with NMOSD with an ARR of<1 than in those with NMOSD with an ARR of ≥1, which was positively correlated with CXCL13. Lastly, the frequency of the Tfh precursor cells decreased in the spleen of keyhole limpet haemocyanin-stimulated animals following GUDCA intervention. These findings significantly broaden our understanding of Tfh cells and CXCL13 in NMOSD. Our data also reveal the potential mechanism of intestinal microbiota and metabolites involved in NMOSD recurrence.

## Introduction

Neuromyelitis optica spectrum disorder (NMOSD) is an autoimmune disease of the central nervous system that repeatedly involves the optic nerve and spinal cord and is mediated by aquaporin-4 (AQP4) antibodies. The combination of AQP4-Ab with AQP4 expressed on astrocytes of the blood–brain barrier causes complement-dependent cytotoxicity and neutrophil, eosinophil, and cytokine infiltration. Blood-brain barrier destruction results in oligodendrocyte death, myelin loss, and neuronal damage.

Recent data have indicated that the germinal centre (GC) may be a pathogenic hotspot for autoantibody production in autoimmune diseases. In particular, circulating Tfh cells that are derived from GC-Tfh cells shuffle between the peripheral blood and lymphoid tissues (1), and play an important role in the differentiation of B cells into memory B and plasma cells, promoting pathogenic autoantibody production, clinical symptom onset, continued immune responsiveness, and eventually irreversible tissue damage (2). Tfh cells function through chemokine CXC receptor 5 (CXCR5), programmed cell death protein-1 (PD-1), cytokine interleukin-21 (IL-21), and C-X-C motif ligand 13 (CXCL13). Multiple studies have shown that Tfh cells expand in the peripheral blood of humans of systemic autoimmune diseases, including rheumatoid arthritis (3), primary Sjogren’s syndrome (4), and immunoglobulin (IgG)-4 related disease (5). Recently, Tfh cells were demonstrated to be reportedly involved in the recurrence of neuroimmune diseases, such as multiple sclerosis (6) and NMOSD (7).

In general, the intestinal microbiota of patients with NMOSD are characterised by lower *Clostridium*, *Parabacteroides*, *Oxalobacter*, and *Burkholderia* and higher *Streptococcus*, *Alistipes*, *Haemophilus*, *Veillonella*, *Butyricimonas*, and *Rothia* abundance than in healthy controls (8, 9). In a previous study, not only did the patients with NMOSD have significantly lower faecal butyrate levels, but the patients with lower Expanded Disability Status Scale (EDSS) scores also showed a major reduction in the butyrate levels (9). The anti-inflammatory effect of short-chain fatty acids is not limited to the intestinal tract; it also increases the Treg level and inhibits Th17 cell differentiation (10). However, limited information is available on the effects of the gut microbiota and metabolites on Tfh and NMOSD recurrence.

Therefore, we assessed the Tfh cell number and frequency in patients with NMOSD with low and high annual recurrence rates (ARRs). We then investigated the effects of the metabolites on the immune system in keyhole limpet haemocyanin (KLH)-stimulated animals. We aimed to provide valuable insights into the causal mechanisms underlying the possible clinical effects of intestinal microbiota and metabolites.

## Materials and Methods

### Clinical Study Design and Population

A total of 109 participants, including 59 patients and 50 age- and sex-matched healthy controls, were enrolled in this study from October 2019 to July 2021 at the Third Affiliated Hospital of Sun Yat-Sen University in southern China. The diagnosis of NMOSD was established based on the International Panel on NMO Diagnosis 2015 criteria (11) and AQP4-IgG seropositivity. We included patients according to the following inclusion criteria: (1) initiation of immunosuppressive treatment as a first-line therapy within 3 years following disease onset and (2) at least 3 months of follow-up. We excluded patients according to the following exclusion criteria: (1) EDSS score ≥ 6.0, (2) body mass index< 20 kg/m^2^, (3) a history of cardiovascular or renal disease and other autoimmune diseases, and (4) age< 18 years. The following data were collected from the participants’ medical records at the baseline visit: demographics (including age, race/ethnicity, sex, weight, and height), past and present diet attempts, serologic status, date of disease onset (first attack), EDSS scores, and modified Rankin Scale scores. NMOSD data collection included a history of recurrence, past/current immunosuppressive therapies, disease duration, medication history, and reasons for treatment discontinuation. Disease onset was defined as the first recurrence of NMOSD. Attack and recurrence were defined as new symptoms that occurred within at least 24 h and were associated with new magnetic resonance imaging lesions. The ethical committee of the Third Affiliated Hospital of Sun Yat-sen University of Medical Sciences approved the research proposal in 10-15-2019. We have registered our trial before the first participant was enrolled in the clinical trial with a project identification code NCT04101058 that are reported in manuscripts (More information about trial registration see https://register.clinicaltrials.gov/prs/app/action/).

Of the 59 patients with NMOSD, serum samples were collected to measure the cytokine levels, and peripheral blood mononuclear cells (PBMCs) were collected for immunological analysis from the 46 patients, who also provided faecal samples for microbiome analysis and metabolomics. These 46 patients were further categorised into two groups based on the ARR: low ARR group (n = 37, ARR< 1) and high ARR group (n = 9, ARR ≥ 1) (**Table 1**). All the 50 age- and sex-matched healthy controls only supplied stool samples for the microbiome analysis.

**Table 1:** Baseline characteristics of the study population.

	Low recurrence rate	High recurrence rate	*p*-value (P3 vs. P2)
N	49	10	
Faecal sample	46	10	
Female, n (%)	93.9%	100%	
Age, years	43.0	47.5	0.568
BMI, kg/m^2^	22.3	23.9	0.202
Work (2), n (%)	33 (84.6%)	8 (80.0%)	0.659
High-oil diet, n (%)	5 (12.8%)	1 (10.0%)	0.781
High-salt diet, n (%)	11 (38.3%)	2 (20.0%)	0.315
Smoker, n (%)	2 (5.13%)	0 (0.0%)	1
Drink alcohol, n (%)	2 (5.13%)	1 (5.3%)	1
Sports frequency (1, 2) (%)	26 (66.7%)	4 (40.0%)	0.384
Sports strength (1, 2) (%)	7 (17.9%)	0 (0.0%)	0.412
AQP4-IgG, n (%)	49 (100%)	10 (100%)	–
ARR, mean	0.4	1.0	≤0.001
Course of disease, years	6	1.5	0.023
EDSS, mean	3.0	2.75	0.955
mRS, n (%)			0.907
0	2 (5.13%)	1 (10.0%)	
1	26 (66.7%)	7 (70.0%)	
2	9 (23.1%)	2 (20.0%)	
3	2 (5.13%)	0 (0.00%)	
Immunosuppressant			0.419
Azathioprine	11 (22.4%)	2 (20.0%)	
Mycophenolate mofetil	23 (46.9%)	7 (70.0%)	

ARR, annual recurrence rate; EDSS, Expanded Disability Status Scale; mRS, modified Rankin Scale.

### Microbiome Analysis

Faecal DNA was isolated using the QIAamp Fast DNA stool Mini Kit (Qiagen, Cat# 51604), and the V3–V4 region of the 16S rRNA bacterial gene was amplified with barcoded specific bacterial primers: forward primer 5′- ACTCCTACGGGAGGCAGCA-3′ and reverse primer 5′-GGACTACHVGGGTWTCTAAT-3′. The 16S rDNA was polymerase chain reaction (PCR) amplified using Q5^®^ High-Fidelity DNA Polymerase (M0491, NEB, USA). The PCR amplicons were quantified using the MiSeq Reagent Kit v3 (MS-102-3003, Illumina Inc., USA) in a MiSeq-PE250 sequencer (Illumina) based on standard protocols. The 16S rDNA amplicon data were analysed using a customised QIIME2 software pipeline (https://qiime2.org). The readings were then processed using the quantitative insights into microbial ecology (QIIME2) analysis. The taxonomy assignment was based on 97% clustered operational taxonomic units of the Greengenes v13.8 database using the naive Bayesian classifier.

### Stool Metabolomics

Faecal metabolites were extracted using 50% methanol buffer. Faecal samples (20 μL) were added to 120 μL of pre-cooled 50% methanol, vortexed for 60 s, incubated at 4°C for 10 min, incubated at -20°C for 60 min, centrifuged at 4,000 × g at 4°C for 15 min, transferred to another tube, and then analysed by liquid chromatography-mass spectrometry (LC-MS). Additionally, pooled quench cooled samples were prepared by mixing 10 μL of each extraction mixture. These samples were then processed using an LC-MS system according to the manufacturer’s instructions. An LC-MS triple quadrupole mass spectrometer (Shimadzu, LCMS-8050) equipped with LabSolutions was used to collect the primary and secondary mass spectrometry data. The electrospray ionisation ion source parameters for the negative ion mode were set as follows: nebulising gas temperature, 300°C; nebulising gas flow, 3 L/min; heating gas flow, 13 L/min; sheath gas temperature, 350°C; DL temperature, 250°C; heating module temperature, 400°C; and sheath gas flow, 7 L/min. BAs were detected using LabSolutions LCMS software (Shimadzu) to perform peak extraction, peak integration, area calculation on the original file, and quantification using a standard curve.

### Measurement of Cytokines

To measure the cytokine levels, blood samples were centrifuged at 2,500 g for 10 min, and the sera were stored at −80°C. The sera were probed for the following 22 markers: interferon-gamma, IL-1beta, IL-10, IL-13, IL-17A, IL-21, IL-6, IL-7, IL-8, nerve growth factor-beta, tumour necrosis factor, vascular endothelial growth factor A, APRIL, B cell-activating factor receptor, CXCL13, granulocyte colony-stimulating factor, macrophage migration inhibitory, interleukin-1 receptor antagonist, metalloproteinase (MMP)-2, MMP-3, MMP-8, MMP-9. The Cytokine/Chemokine/Growth Factor Convenience 45-Plex Human kit (Thermo Fisher Scientific) was allowed to warm at 15°C for 2 h. All the steps were performed according to the manufacturer’s recommendations.

### Animals

Female BALB/c mice aged 5–6 weeks (15–18 g) were purchased from the Laboratory Animal Center. This study complied with all the relevant ethical regulations and was approved by the Ethics Committee of the South China Agricultural University (ethical number: 2021B002). Animals were housed under specific pathogen-free conditions and maintained over a 12-h light/dark cycle with free access to food and water. Mice received an injection of KLH in both the underarms and groin (100 µg/0.2 mL/site), and the control groups were treated with equal amounts of saline. Mice were then divided into three groups (n = 6 animals per group): control, KLH, and KLH+glycoursodeoxycholic acid (GUDCA) acid groups. Each experimental group was separately gavaged with 50 mg/kg body weight of GUDCA for 4 weeks. The control and KLH groups were treated with equal amounts of saline. After treatment, ileum, spleen, and lymph node samples were collected to analyse the Tfh cell phenotype by fluorescence-activated cell sorting.

### Flow Cytometry

FITC-conjugated anti-CD4 (human), BV510-conjugated anti-CXCR5 (human), PE-conjugated anti-CCR7 (human), AF700-conjugated anti-CD4 (mouse), APC-conjugated anti-CXCR5 (mouse), PE-conjugated anti-CCR7 (mouse), and FITC-conjugated-anti-PD-1 (mouse) were purchased from BioLegend (San Diego, CA, USA). PBMCs were separated and frozen at −80°C until phenotypic analysis was performed according to standard protocols. Samples were acquired using a BD LSR II flow cytometer (BD Bioscience) and analysed using FlowJo.

### Immunofluorescence

Immunostaining was performed as previously described. For immunostaining, the gut sections were fixed, blocked, and incubated with primary antibodies against CCR7 (BioLegend, 1:200), CXCR5 (BioLegend, 1:200), and PD-1 (BioLegend, 1:200) at 4°C overnight. After washing, the sections were co-stained with 2 μg/mL 4′,6-diamidino-2-phenylindole (nucleus) for 5 min. Fluorescence images were acquired using a confocal microscope (Leica SP8) with a 20× objective.

### Statistical Analysis

All the statistical analyses were performed using the Statistical Package for the Social Sciences software version 19.0; the data are presented as mean values with standard deviation. The test of normal distribution was performed before the Student’s *t*-test and analysis of variance (ANOVA). Multiple comparisons were performed using one-way ANOVA or Kruskal–Wallis tests between the different groups. The Shapiro-Wilk test was used to assess normality. Inflammatory cytokines levels were log_10_-transformed when analysed to meet the normal assumption. The correlation between the two variables was assessed using the Spearman rank test. For all analyses, a *p*-value of<0.05 was considered to indicate statistical significance. Correlations between each of the pair of datasets were computed using Pearson correlation coefficients, and visualisations were generated in R (v4.0.0).

## Results

### CXCL13 Was Significantly Lower in Patients of NMOSD With a Low ARR

**Figure 1:**
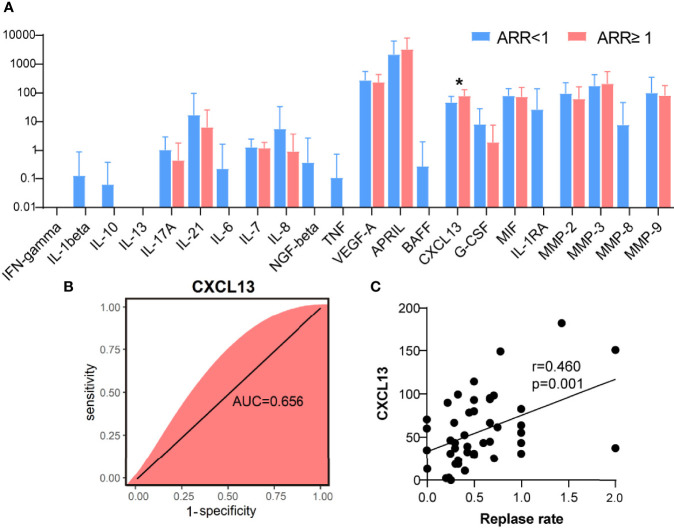
C-X-C motif ligand 13 (CXCL13) as a biological marker for neuromyelitis optica spectrum disorder recurrence. **(A)** Quantification of inflammatory cytokines between two groups of patients with NMOSD (N = 37 in ARR<1 patients, N = 9 in ARR ≥ 1 patients). **(B)** Random forest analysis showed the CXCL13 could discriminate the two groups based on the area under the curve (0.656). **(C)** Using the CXCL13, a relatively poor correlation was achieved with Spearman’s correlation analysis (*r* = 0.460). ARR, annual recurrence rate. *p < 0.05.

The potential value of serum inflammatory cytokine markers was investigated in the diagnosis of NMOSD (**Table 2**). CXCL13 was significantly different between the two groups (51.82 vs. 78.88, *p =* 0.019, **Figure 1A**). The individual simplified signature could discriminate between the two groups based on the area under the curve (AUC) (CXCL13 [AUC = 0.656], **Figure 1B**). We also aimed to explore the correlations between these discriminative inflammatory factors and ARR to identify markers associated with disease recurrence. Spearman’s rank correlation analysis showed that CXCL13 (*r* = 0.460, *p* = 0.001) was positively correlated with NMOSD recurrence (**Figure 1C**).

**Table 2:** Quantitative data of inflammatory cytokines in NMOSD patients.

	Low recurrence rate (range)	High recurrence rate (range)	*p*-value
N	37	9	
IFN-gamma	0.000	0.000	1
IL-1beta	0.12 (0.00–4.69)	0.000	1
IL-10	0.06 (0.00–1.98)	0.000	1
IL-13	0.000	0.000	1
IL-17A	1.06 (0.00–5.78)	0.45 (0.00–4.07)	0.367
IL-21	16.90 (0.00–474.73)	6.41 (0.00–57.67)	0.799
IL-6	0.23 (0.00–8.65)	0.000	1
IL-7	1.30 (0.06–1.62)	1.20 (0.00–1.35)	0.647
IL-8	5.599 (0.00–168.43)	0.93 (0.00–8.38)	0.69
NGF-beta	0.38 (0.00–14.15)	0.000	1
TNF	0.11 (0.00–3.94)	0.000	1
VEGF-A	274.7 (17.64–1284.32)	235.7 (0.00–600.4)	0.792
APRIL	2163 (0.00–9740.55)	3240 (0.00–9880.43)	0.448
BAFF	0.28 (0.00–10.51)	0.00	1
CXCL13	51.82 (0.00–149.08)	78.88 (30.89–181.78)	0.019*
G-CSF	8.09 (0.00–108.58)	1.91 (0.00–17.16)	0.357
MIF	80.32 (49.3–93.6)	73.79 (0.00–218.56)	0.124
IL-1RA	26.48 (0.00–650.43)	0.00	
MMP-2	96.15 (0.00–130)	61.04 (0.00–294.62)	0.355
MMP-3	174.7 (0.00–334)	208.13 (0.00–1053.88)	0.686
MMP-8	7.76 (0.00–226.57)	0.00	1
MMP-9	100.00 (0.00–1340.75)	80.51 (0.00–244.47)	0.572

*p < 0.05.

### Tfh Cells Were Positively Correlated With NMOSD Recurrence

**Figure 2:**
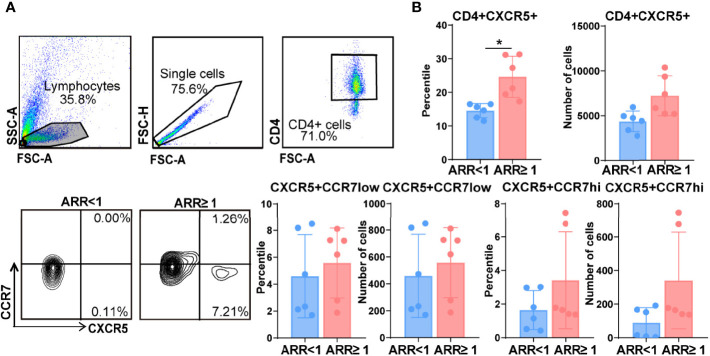
Increased frequency of follicular helper T (Tfh) cells in patients with neuromyelitis optica spectrum disorder (NMOSD) with a high annual recurrence rate. **(A)** Comparison of the frequencies of circulating Tfh cells in patients with two groups patients with NMOSD. Representative expressions of CXCR5+CD4+ T cells, CXCR5+CCR7^low^ Tfh progenitors, and CXCR5+CCR7^hi^ Tfh were detected by flow cytometry. **(B)** Flow cytometric analysis of the different phenotypes of Tfh. N = 6, ^*^p < 0.05. ARR, annual recurrence rate.

The frequency of circulating CXCR5^+^CD4^+^ Tfh, CXCR5^+^CCR7^low^ Tfh precursor, and CXCR5+CCR7^hi^ resting Tfh cells were analysed using flow cytometry. As shown in **Figure 2A**, the percentage of CD4^+^CXCR5^+^ T cells was significantly higher in the peripheral blood of patients with NMOSD with an ARR of ≥ 1 than in those with an ARR of <1 (24.6% vs. 14.5%, *p* < 0.01). Moreover, the percentage of CXCR5^+^CCR7^hi^ Tfh cells among CD4^+^ T cells was higher in patients with NMOSD having an ARR of ≥ 1 than in those with NMOSD having an ARR of <1 (3.4.% vs. 1.6%, *p* = 0.07); however, owing to the large individual difference of patients, there was no significant difference between these patients. There were no significant differences in the frequency or number of CXCR5^+^CCR7^low^ T cells between the two groups of patients, although both showed higher levels in the patients with an ARR of ≥ 1 than in the patients with an ARR of<1 (**Figures 2A, B**). Therefore, we speculated that more CXCR5^+^CCR7^hi^ Tfh cells among CD4^+^ T cells in the patients with NMOSD having a higher ARR could explain the higher CD4^+^CXCR5^+^ T cells in patients with higher ARR, although there was no significant difference between these patients.

### Decreased CXCL13 Level Was Associated With GUDCA

**Figure 3:**
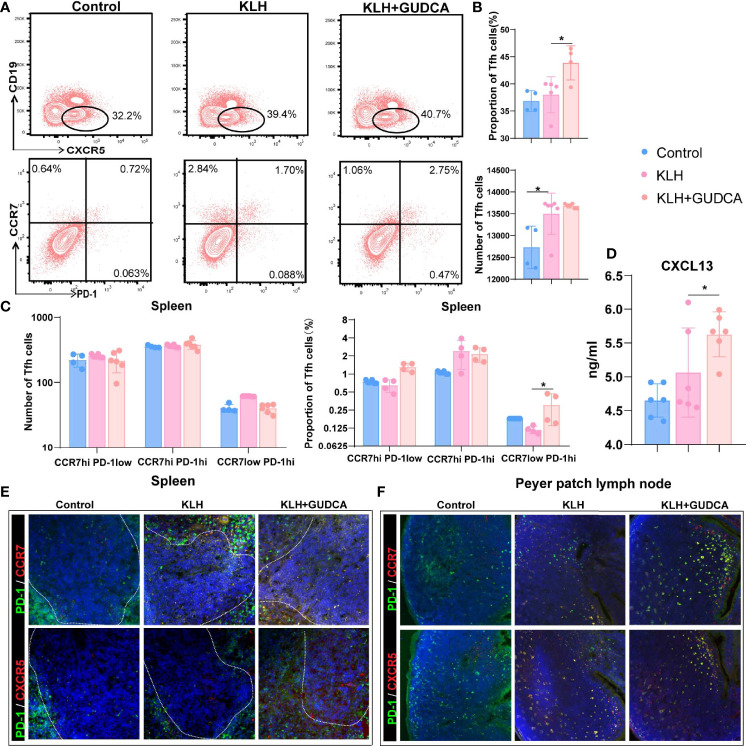
Glycoursodeoxycholic acid administration inhibited the T follicular helper (Tfh) cells in the spleen in KLH-stimulated animals. **(A)** The differentiated plasma cells from B cells were detected using flow cytometry in KLH-stimulated animals. **(B)** Flow cytometric analysis of CD4+CD19-CXCR5+ T cells. **(C)** Flow cytometric analysis of CCR7^hi^PD-1^low^ resting Tfh, CCR7^hi^PD-1^hi^-activated Tfh, and CCR7^low^PD-1^hi^ Tfh cells. **(D)** Expression of CXCL13 in peripheral blood of KLH-stimulated animal using enzyme-link immunosorbent assay. **(E, F)** Representative immunofluorescence image of CXCR5+PD-1^hi^ Tfh and CCR7+PD-1^hi^ Tfh cells in the spleen and Peyer’s patch lymph node of the KLH-stimulated animal. N = 6, ^*^p < 0.05. KLH, keyhole limpet haemocyanin; GUDCA, glycoursodeoxycholic acid.

The number of CXCR5+CD4+ T cells was significantly higher in the KLH group than in the GUDCA group (*p* < 0.001, **Figures 3A, B**). Further, there was an increasing trend of CXCR5^+^CD4^+^ Tfh cell count in the GUDCA group (*p* = 0.02, **Figures 3A, B**). In addition, we evaluated the frequency of CD19^low^CXCR5^+^ Tfh cells, the frequency of CCR7^hi^PD-1^low^ resting Tfh and CCR7^hi^PD-1^hi^-activated Tfh cells. Tfh cell count did not change significantly, whereas CCR7^low^PD-1^hi^ Tfh precursor cells in KLH mice tended to increase following GUDCA supplementation compared with those in the KLH group (**Figures 3A, C**). In addition, a significant increase in the serum CXCL13 level was observed in the GUDCA group compared with that in the KLH group (**Figure 3D**), which supports the immunomodulatory function of GUDCA.

We then analysed the distribution of splenic (PD-1^+^ and CXCR5^+^/CCR7^+^ double-positive) Tfh cells with immunohistochemical double staining. Splenic PD-1^+^CCR7^+^ and PD-1^+^CXCR5^+^ Tfh cells were localised in the T-B cell zone in the control group, whereas they were distributed in the GC-B-cell follicles in the KLH and GUDCA groups (**Figure 3E**). There were barely PD-1^+^CCR7^+^ and PD-1^+^CXCR5^+^ positive Tfh cells in the intestinal Peyer’s patch lymph node of the control group, which accumulated around the margin of Peyer’s patch lymph node (**Figure 3F**), supporting the notion that GUDCA supplied a permissive environment for Tfh cell generation.

### Disease Relapse Was Associated With Significant Variations of the Gut Microbiome

**Figure 4:**
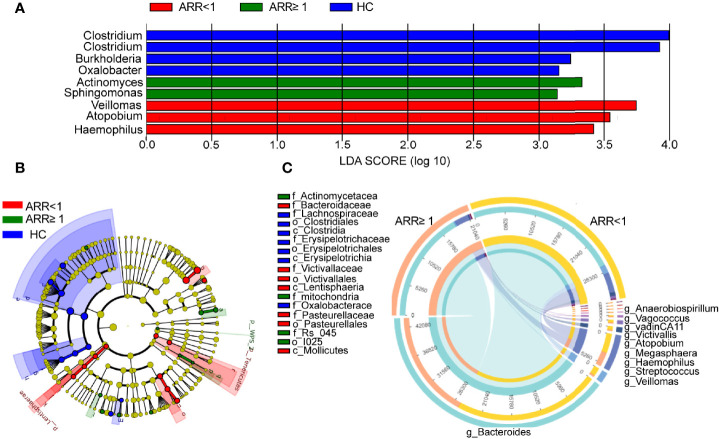
Recurrence-specific changes in bacterial diversities and taxonomic signatures. **(A)** Species were predicted using linear discriminant analysis effect size (LEfSe) at the genus level between two groups patients. **(B)** Cladogram of the LEfSe analysis. **(C)** Correlation detection of ARR-associated gut bacteria in patients with NMOSD. ARR, annual recurrence rate; HC, Healthy controls.

Next, we analysed the changes in the stool microbiota of patients with NMOSD using 16S rRNA gene sequencing at the genus taxonomic rank levels (**Figure 4A**). Patients with NMOSD having a high ARR were accompanied by a relatively higher abundance of *Actinomyces* and *Sphingomonas* but a low abundance of *Veillomas*, *Atopobium*, and *Haemophilus* (**Figure 4A**). A higher stool relative abundance of *Actinomycetaceae* (such as *Actinomyces*) and *Mitochondria* predicted a high ARR in these 59 patients, whereas *Bacteroidaceae* (such as *Bacteroides*), *Victivallaceae* (such as *Victivallis*), or *Pasteurellaceae* family members were associated with an optimistic prognosis (**Figure 4B**). Interestingly, *Vagococcus*, *Anaerobiospirillum*, *Stenotrophomonas*, *Veillomas*, *Megasphaera*, *Atopobium*, *VadinCA11*, *Victivallis, Haemophilus* and *Bacteroides* were negatively correlated with ARR in NMOSD (**Figure 4C**). Hence, NMOSD recurrence is associated with the local microbiome.

### GUDCA Increased the Circulating Tfh Cell and CXCL13 Levels in Mice

**Figure 5:**
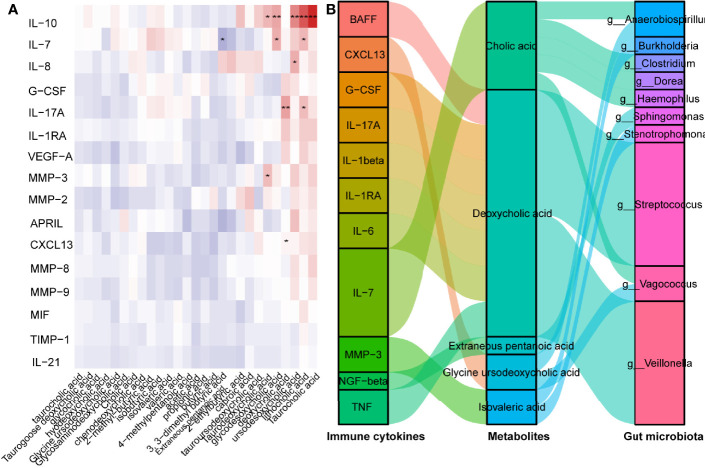
GUDCA increased circulating Tfh cell and CXCL13 levels in mice. **(A)** Correlation between inflammatory factors and metabolites were analysed by Spearman’s correlation. Significant changes are denoted as follows: ^**^p < 0.01 and ^*^p < 0.05. **(B)** Spearman’s correlation of serum inflammatory cytokines with gut metabolites, gut metabolites and gut bacterial species.

Subsequently, we concentrated on the association between CXCL13 and gut microbiota-derived metabolites. Pearson’s correlation analysis revealed that GUDCA was positively correlated with the CXCL13 levels (**Figures 5A, B**). A positive correlation between the Taurocholic acid (TCA), LCA ursodesoxycholic acid (UDCA), glycodesoxycholic acid (GDCA) and Taurodeoxycholic acid (TDCA) and the IL-10 levels (*p* < 0.001, *p* < 0.001, *p* < 0.001, *p* = 0.006 and *p* = 0.033, respectively) and a positive correlation between the DCA and LCA, and the IL-17 concentration (*p* = 0.005 and *p* =0.039, respectively) in NMOSD were also observed (**Figure 5A**). *Anaerobiospirillum* and *Vagococcus* were negatively correlated with GUDCA levels (**Figure 5B**). Using the Virtual Metabolic Human database, we identified that reduced *Veillomas* and *Haemophilus* abundance in Spatients with NMOSD having a high ARR may contribute to decreased cholic acid (CA) and deoxycholic acid (DCA) concentration, which are the metabolites of GUDCA. These data indicate that *Anaerobiospirillum* and *Vagococcus* were the most substantial gut microbiota influencing the levels of GUDCA in the hosts, whereas *Veillomas* and *Haemophilus* were the most substantial gut microbiota in converting GUDCA to DCA (**Figure 5B**).

## Discussion

Our findings indicate that the gut microbiota play a crucial role in governing the Tfh immune response induced by CXCL13 in patients with NMOSD. In this study, we analysed the interplay between the stool metabolites and systemic immunomodulation and found significantly downregulated levels of CXCL13 in patients with NMOSD with low ARR. CXCL13 was negatively correlated with GUDCA, which was positively correlated with *Vagococcus and Anaerobiospirillum* abundance, which were negatively correlated with ARR in NMOSD, eliciting CXCL13-induced Tfh cell immune responses possibly dependent on GUDCA and gut microbiota. In this study, we confirmed an increase in the CXCR5+CD4+ T cells in patients with NMOSD with a high ARR. Certainly, serum CXCL13 level reflects the priming and activation of Tfh cells in the spleen. In contrast, GC-Tfh is one of the main producers of plasma CXCL13. There is an increasing number of studies on the Tfh cells in NMO or NMOSD. The level of chemokine CXCL13 in the cerebrospinal fluid of patients with NMO is related to the degree of NMO-related nerve defect (7), and patients with recurrence tend to have higher levels of CXCL13 in the serum and cerebrospinal fluid (CSF). The CXCR5 levels in the serum and CSF of patients with NMO are elevated (7). The administration of rituximab (a B-cell depletion therapy) can dramatically reduce the Tfh ratio and re-establish the Tfh subpopulation (12). In summary, the Tfh cells are associated with the disease activity of NMOSD, and could be potential immune markers for disease monitoring, and provide a new direction for NMOSD immunotherapy (13).

Studies have also shown that memory Tfh cells in the circulation express the same surface molecules as effector Tfh cells and can effectively assist B cells in immune responses (14). Therefore, memory Tfh cells can be used as marker cells to monitor the immune activation status of autoimmune diseases. Previous study showed that circulating memory Tfh cells, especially CCR7^+^ICOS^+^ memory Tfh cells, may be associated with the relapse of MS and the numbers of circulating memory Tfh cells significantly decreased in the remitting stage (15). Among the circulating memory Tfh cells, CCR7^+^ memory Tfh cells can migrate into B cell follicles and promote humoral responses (16, 17), and activated CCR7^-^PD-1^+^ memory Tfh cells in the secondary lymphoid tissues also enhance the humoral responses, associated with the development of autoimmune diseases (18). Accordingly, in patients with systemic lupus erythematosus (SLE) and rheumatoid arthritis (RA), increased levels of the CCR7^lo^PD-1^hi^ subset correlated with elevated autoantibody profiles and more severe disease activities (18). Intriguingly, our results indicated significantly increased numbers of CCR7^-^PD-1^+^ memory Tfh cells in GUDCA-treated KLH mice compared to KLH mice, and the CXCL13 levels were significantly increased in mice sero, Therefore, CXCL13 may participate in the migration of CCR7^+^ and CCR7^+^ICOS^+^ memory Tfh cells into the secondary lymphoid organs, and consequently, the activated CCR7^-^PD-1^+^ memory Tfh cells could enhance the humoral responses associated with the relapse of NMOSD.

Another cytokine, IL-21, also plays a critical role in the survival of Tfh cells and the survival, proliferation, and differentiation of GC B cells. Studies have shown that the frequencies of circulating CD4^+^CXCR5^+^PD-1^+^ T cells and serum IL-21 were higher in patients with NMOSD than in those with MS (19). After glucocorticoid therapy, CD4^+^CXCR5^+^PD-1^+^ T cell count and serum IL-21 levels decreased, suggesting that the Tfh cells and IL-21 are associated with the disease activity of NMOSD (19). A recent study found that the circulating memory Tfh cells (ICOS^+^, CCR7^−^, CCR7^−^, ICOS^+^, CCR7^+^, and CCR7^+^ICOS^+^ Tfh cells) and serum and cerebrospinal fluid IL-21 levels in patients with NMO/NMOSD significantly increased (7). The proportion of CCR7^-^ and CCR7^-^ICOS^+^ memory Tfh cells is positively correlated with the ARR, plasma IL-21 level, and AQP-4 antibody levels (7). The proportion of CCR7^+^ and CCR7^+^ ICOS^+^ memory Tfh cells is positively correlated with the number of white blood cells and IL-21 in CSF. After hormone therapy, the levels of CCR7^-^ICOS^+^, CCR7^+^ICOS^+^ Tfh cells, and IL-21 reduced in patients with complete remission (7). Hence, IL-21 may participate in the development and relapse of NMO/NMOSD. Although significantly different levels of IL-21 between two groups were not observed in the present study, IL-21 could be utilised as a therapeutic target in NMOSD.

We found that the increased faecal levels of GUDCA induced Tfh activation. Primary BAs in the gut are derived from cholesterol and are synthesised in the liver. BAs are primarily synthesised by the rate-limiting enzyme cholesterol 7α-hydroxylase from the cholesterol in the liver, conjugated with either glycine or taurine (20). Bile salt deconjugation is catalysed by bile salt hydrolase (BSH) and generates free BAs (21), including mainly CDCA and trihydroxy BA CA. The gut microbiome is the only source of enzymes capable of generating hydrophobic SBAs. *Clostridium* and *Desulfovibrio*, which encode the bile acid-inducible operon, convert the host CA to DCA and CDCA and UDCA to LCA (22). In the present study, we found that the *Veillomas* and *Haemophilus* showed the highest increase in patients with NMOSD having an ARR of<1, which could explain the lower levels of GUDCA in the stool. In particular, *Veillomas* and *Haemophilus* spp. were downregulated in patients with NMOSD having a high ARR and negatively correlated with the serum GUDCA levels. The relative abundance of *Veillomas* increased in marathon runners, and inoculation of Veillonella into mice significantly increased the running time (23). Previous studies have reported that the abundance of *Veillonella* increased in patients with cirrhosis (24), whereas the levels of intestinal BAs in patients with liver cirrhosis are frequently insufficient (25). *Haemophilus* displayed a negative correlation with common indicators of dyslipidaemia, such as total cholesterol and high-density lipoprotein cholesterol (26). *Haemophilus* has also been reported to have a negative association with DCA (27). However, the levels of BAs were similar in both groups of NMOSD, ruling out that the discriminant bacteria observed between the two groups were caused by these metabolites. These investigations on the intestinal symbiotic bacteria provide evidence for a precise understanding of the complex interactions between the gut microbiota and host BA metabolites. Our data suggested that the decreased CXCL13 level was associated with GUDCA, CCR7^low^PD-1^hi^ Tfh precursor cells in the KLH mice tended to increase following GUDCA supplementation, showing that the levels of CXCL13 and Tfh precursor cells were correlated with the metabolite GUDCA. Meanwhile, *Veillomas* and *Haemophilus* showed the highest increase in patients with NMOSD having an ARR of<1, which could explain the lower levels of GUDCA in the stool. Therefore, our data showed that the recurrence rate correlated with the gut microbiota and their metabolite in NMOSD, whereas the CXCL13-induced activation of the Tfh cells was the possible mechanism.

In addition, recent reports have confirmed that the intestinal microbiota is essential for the local activation of Tfh cells. Immunogenic commensals are distinguishable from tolerogenic bacteria and trigger migratory DCs to release IL-1β and IL-12p70, thereby increasing bacterial or self-antigen-specific Tfh cells or IgG2b responses (28). Various microbial contents can trigger Tfh cell responses, specifically bacterial RNA, which can induce IL-1β-dependent differentiation of Tfh cells and GC B cells (29). After mucosal inoculation with inactivated enterotoxigenic *Escherichia coli*, activated ICOS^+^ Tfh cells were recirculated in the blood and represented mucosal memory B cell responses (30). Whether antigen-specific Tfh cells are present in NMOSD remains to be defined. Further experiments are needed to address the gut microbiota to detect whether there is a Tfh-specific recognition motif.

In our study, we demonstrated that the gut microbiota and their metabolite correlated to the NMOSD relapse *via* CXCL13-induced activation of Tfh cells. In addition, there was a significant correlation between the multiple microbe metabolites and IL-10 and IL-17A in our study. Previous studies have revealed that the interaction between hosts and their gut bacteria can regulate the host immunological IL10/IL17A homeostasis *via* the BAs. Song et al. have reported that genetic abolition of the BA metabolic pathways in individual gut symbionts significantly decreases this Treg cell population. Restoration of the intestinal BA pool increases the colonic RORγ+ Treg cell counts and ameliorates host susceptibility to inflammatory colitis *via* BA nuclear receptors (31). Campbell et al. found that the secondary bile acid 3β-hydroxydeoxycholic acid (isoDCA) increased the Foxp3 induction by acting on dendritic cells (DCs) to diminish their immunostimulatory properties. Ablating one receptor, the FXR, in DCs enhanced the generation of Treg cells and imposed a transcriptional profile similar to that induced by isoDCA (10). Moreover, IL10/IL17A homeostasis reportedly participates in the pathogenesis of NMOSD (32). Therefore, more research is warranted to better explain the role of gut microbiota and their metabolite in the pathogenesis of NMOSD.

This study has some limitations. Although we noted the accumulation of Tfh cells in the lymph follicle-like structure in the gut and spleen, the origin of Tfh cells remains unclear. We also noted that the number of participants in our cross-sectional analysis was relatively small. We can only verify the role of GUDCA in Tfh activation in the animal experiments. However, there is no direct evidence of whether the gut microbiota affects Tfh activation. In fact, it is not clear if different immunosuppressive treatments and course of disease between the groups may affect Tfh activation.

## Conclusions

In the present study, we demonstrated that the recurrence of NMOSD is correlated with Tfh cells and CXCL13, which is positively correlated with gut GUDCA. These data broaden our understanding of the mechanism of NMOSD and provide intestinal microbiota as a potential therapeutic target. Finally, we established a gut microbiome–metabolite–Tfh-CXCL13 system to predict the recurrence of NMOSD.

## Data Availability Statement

The datasets presented in this study can be found in online repositories. The names of the repository/repositories and accession number(s) can be found in the article/**Supplementary Material**.

## Ethics Statement

The studies involving human participants were reviewed and approved by the medical ethics committee of the Third Affiliated Hospital of Sun Yat-sen University. The patients/participants provided their written informed consent to participate in this study. The animal study was reviewed and approved by Medical Ethics Committee of The Third Affiliated Hospital of Sun Yat-sen University.

## Author Contributions

Conceptualisation, WQ and AK. Methodology, XC. Software, XC. Validation, CL. Formal analysis, XC. Investigation, LZ. Data curation, XZ and YC. Writing—original draft preparation. XC. Writing—review and editing, ZL and LZ. Visualisation, SS. Supervision, YZ. Project administration, XC. Funding acquisition, WQ. All authors have read and agreed to the published version of the manuscript.

## Funding

Financial support for the research was provided by the National Natural Science Foundation of China (grants. 82001284, 82071344, and 82101418).

## Conflict of Interest

The authors declare that the research was conducted in the absence of any commercial or financial relationships that could be construed as a potential conflict of interest.

## Publisher’s Note

All claims expressed in this article are solely those of the authors and do not necessarily represent those of their affiliated organizations, or those of the publisher, the editors and the reviewers. Any product that may be evaluated in this article, or claim that may be made by its manufacturer, is not guaranteed or endorsed by the publisher.

## References

[1] Zhang X Ing S Fraser A Chen M Khan O Zakem J. Follicular Helper T Cells: New Insights Into Mechanisms of Autoimmune Diseases. Ochsner J (2013) 13:131–9. PMC3603176 23531878

[2] Simpson N Gatenby PA Wilson A Malik S Fulcher DA Tangye SG. Expansion of Circulating T Cells Resembling Follicular Helper T Cells is a Fixed Phenotype That Identifies a Subset of Severe Systemic Lupus Erythematosus. Arthritis Rheum (2010) 62:234–44. doi: 10.1002/art.25032 20039395

[3] Ma J Zhu C Ma B Tian J Baidoo SE Mao C. Increased Frequency of Circulating Follicular Helper T Cells in Patients With Rheumatoid Arthritis. Clin Dev Immunol (2012) 2012:1–7. doi: 10.1155/2012/827480 PMC3357937 22649468

[4] Szabo K Papp G Barath S Gyimesi E Szanto A Zeher M. Follicular Helper T Cells may Play an Important Role in the Severity of Primary Sjögren’s Syndrome. Clin Immunol (2013) 147:95–104. doi: 10.1016/j.clim.2013.02.024 23578551

[5] Kubo S Nakayamada S Zhao J Yoshikawa M Miyazaki Y Nawata A. Correlation of T Follicular Helper Cells and Plasmablasts With the Development of Organ Involvement in Patients With Igg4-Related Disease. Rheumatol (United Kingdom) (2018) 57:514–24. doi: 10.1093/rheumatology/kex455 29253269

[6] Holm Hansen R Højsgaard Chow H Sellebjerg F Rode von Essen M. Dimethyl Fumarate Therapy Suppresses B Cell Responses and Follicular Helper T Cells in Relapsing-Remitting Multiple Sclerosis. Mult Scler J (2019) 25:1289–97. doi: 10.1177/1352458518790417 30043661

[7] Fan X Jiang Y Han J Liu J Wei Y Jiang X. Circulating Memory T Follicular Helper Cells in Patients With Neuromyelitis Optica/Neuromyelitis Optica Spectrum Disorders. Mediators Inflamm (2016) 2016:1–13. doi: 10.1155/2016/3678152 PMC4804098 27057097

[8] Cui C Tan S Tao L Gong J Chang Y Wang Y. Intestinal Barrier Breakdown and Mucosal Microbiota Disturbance in Neuromyelitis Optical Spectrum Disorders. Front Immunol (2020) 11:1–15. doi: 10.3389/fimmu.2020.02101 32983166 PMC7492665

[9] Gong J Qiu W Zeng Q Liu X Sun X Li H. Lack of Short-Chain Fatty Acids and Overgrowth of Opportunistic Pathogens Define Dysbiosis of Neuromyelitis Optica Spectrum Disorders: A Chinese Pilot Study. Mult Scler J (2019) 25:1316–25. doi: 10.1177/1352458518790396 30113252

[10] Campbell C McKenney PT Konstantinovsky D Isaeva OI Schizas M Verter J. Bacterial Metabolism of Bile Acids Promotes Generation of Peripheral Regulatory T Cells. Nature (2020) 581:475–9. doi: 10.1038/s41586-020-2193-0 PMC7540721 32461639

[11] Wingerchuk DM Banwell B Bennett JL Cabre P Carroll W Chitnis T. International Consensus Diagnostic Criteria for Neuromyelitis Optica Spectrum Disorders. Neurology (2016) 86:491–2. doi: 10.1212/WNL.0000000000002366 26833940

[12] Nicolas P Ruiz A Cobo-Calvo A Fiard G Giraudon P Vukusic S. The Balance in T Follicular Helper Cell Subsets Is Altered in Neuromyelitis Optica Spectrum Disorder Patients and Restored by Rituximab. Front Immunol (2019) 10:1–7. doi: 10.3389/fimmu.2019.02686 31803192 PMC6877601

[13] Zhao C Li HZ Zhao D Ma C Wu F Bai YN. Increased Circulating T Follicular Helper Cells are Inhibited by Rituximab in Neuromyelitis Optica Spectrum Disorder. Front Neurol (2017) 8:1–9. doi: 10.3389/fneur.2017.00104 28360886 PMC5350120

[14] Helmold Hait S Hogge CJ Rahman MA Hunegnaw R Mushtaq Z Hoang T. TFH Cells Induced by Vaccination and Following SIV Challenge Support Env-Specific Humoral Immunity in the Rectal-Genital Tract and Circulation of Female Rhesus Macaques. Front Immunol (2021) 11:1–21. doi: 10.3389/fimmu.2020.608003 PMC7876074 33584682

[15] Fan X Jin T Zhao S Liu C Han J Jiang X. Circulating CCR7+ICOS+ Memory T Follicular Helper Cells in Patients With Multiple Sclerosis. PloS One (2015) 10:1–14. doi: 10.1371/journal.pone.0134523 PMC4521720 26231034

[16] Morita R Schmitt N Bentebibel SE Ranganathan R Bourdery L Zurawski G. Human Blood CXCR5+CD4+ T Cells Are Counterparts of T Follicular Cells and Contain Specific Subsets That Differentially Support Antibody Secretion. Immunity (2011) 34:108–21. doi: 10.1016/j.immuni.2010.12.012 PMC3046815 21215658

[17] Breitfeld D Ohl L Kremmer E Ellwart J Sallusto F Lipp M. Follicular B Helper T Cells Express CXC Chemokine Receptor 5, Localize to B Cell Follicles, and Support Immunoglobulin Production. J Exp Med (2000) 192:1545–51. doi: 10.1084/jem.192.11.1545 PMC2193094 11104797

[18] He J Tsai LM Leong YA Hu X Ma CS Chevalier N. Circulating Precursor CCR7loPD-1hi CXCR5+ CD4+ T Cells Indicate Tfh Cell Activity and Promote Antibody Responses Upon Antigen Reexposure. Immunity (2013) 39:770–81. doi: 10.1016/j.immuni.2013.09.007 24138884

[19] Yang X Peng J Huang X Liu P Li J Pan J. Association of Circulating Follicular Helper T Cells and Serum CXCL13 With Neuromyelitis Optica Spectrum Disorders. Front Immunol (2021) 12:1–8. doi: 10.3389/fimmu.2021.677190 PMC8316915 34335576

[20] Sayin SI Wahlström A Felin J Jäntti S Marschall HU Bamberg K. Gut Microbiota Regulates Bile Acid Metabolism by Reducing the Levels of Tauro-Beta-Muricholic Acid, a Naturally Occurring FXR Antagonist. Cell Metab (2013) 17:225–35. doi: 10.1016/j.cmet.2013.01.003 23395169

[21] Jones BV Begley M Hill C Gahan CGM Marchesi JR. Functional and Comparative Metagenomic Analysis of Bile Salt Hydrolase Activity in the Human Gut Microbiome. Proc Natl Acad Sci United States America (2008) 105:13580–5. doi: 10.1073/pnas.0804437105 PMC2533232 18757757

[22] Just S Mondot S Ecker J Wegner K Rath E Gau L. The Gut Microbiota Drives the Impact of Bile Acids and Fat Source in Diet on Mouse Metabolism. Microbiome (2018) 6:1–18. doi: 10.1186/s40168-018-0510-8 30071904 PMC6091023

[23] Scheiman J Luber JM Chavkin TA MacDonald T Tung A Pham LD. Meta-Omics Analysis of Elite Athletes Identifies a Performance-Enhancing Microbe That Functions *via* Lactate Metabolism. Nat Med (2019) 25:1104–9. doi: 10.1038/s41591-019-0485-4 PMC7368972 31235964

[24] Chen Y Ji F Guo J Shi D Fang D Li L. Dysbiosis of Small Intestinal Microbiota in Liver Cirrhosis and Its Association With Etiology. Sci Rep (2016) 6:1–9. doi: 10.1038/srep34055 27687977 PMC5043180

[25] Oh TG Kim SM Caussy C Fu T Guo J Bassirian S. A Universal Gut-Microbiome-Derived Signature Predicts Cirrhosis. Cell Metab (2020) 32:878–888.e6. doi: 10.1016/j.cmet.2020.06.005 32610095 PMC7822714

[26] Granado-Serrano AB Martín-Garí M Sánchez V Riart Solans M Berdún R Ludwig IA. Faecal Bacterial and Short-Chain Fatty Acids Signature in Hypercholesterolemia. Sci Rep (2019) 9:1–13. doi: 10.1038/s41598-019-38874-3 30742005 PMC6370822

[27] De Chiara M Hood D Muzzi A Pickard DJ Perkins T Pizza M. Genome Sequencing of Disease and Carriage Isolates of Nontypeable Haemophilus Influenzae Identifies Discrete Population Structure. Proc Natl Acad Sci USA (2014) 111:5439–44. doi: 10.1073/pnas.1403353111 PMC3986186 24706866

[28] Roberti MP Yonekura S Duong CPM Picard M Ferrere G Tidjani Alou M. Chemotherapy-Induced Ileal Crypt Apoptosis and the Ileal Microbiome Shape Immunosurveillance and Prognosis of Proximal Colon Cancer. Nat Med (2020) 26:919–31. doi: 10.1038/s41591-020-0882-8 32451498

[29] Barbet G Sander LE Geswell M Leonardi I Cerutti A Iliev I. Sensing Microbial Viability Through Bacterial RNA Augments T Follicular Helper Cell and Antibody Responses. Immunity (2018) 48:584–98.e5. doi: 10.1016/j.immuni.2018.02.015 29548673 PMC5924674

[30] Cárdeno A Magnusson MK Quiding-Järbrink M Lundgren A. Activated T Follicular Helper-Like Cells are Released Into Blood After Oral Vaccination and Correlate With Vaccine Specific Mucosal B-Cell Memory. Sci Rep (2018) 8:1–15. doi: 10.1038/s41598-018-20740-3 29426881 PMC5807513

[31] Song X Sun X Oh SF Wu M Zhang Y Zheng W. Microbial Bile Acid Metabolites Modulate Gut Rorγ+ Regulatory T Cell Homeostasis. Nature (2020) 577:410–5. doi: 10.1038/s41586-019-1865-0 PMC7274525 31875848

[32] Varrin-Doyer M Spencer CM Schulze-Topphoff U Nelson PA Stroud RM Bruce BA. Aquaporin 4-Specific T Cells in Neuromyelitis Optica Exhibit a Th17 Bias and Recognize Clostridium ABC Transporter. Ann Neurol (2012) 72:53–64. doi: 10.1002/ana.23651 22807325 PMC3405197

