# Supplementary figures and images for: Gut Microbiome and Bile Acid Metabolism Induced the Activation of CXCR5+ CD4+ T Follicular Helper Cells to Participate in Neuromyelitis Optica Spectrum Disorder Recurrence
Source: Front Immunol. 2022 Jan 20;13:827865. doi: 10.3389/fimmu.2022.827865 (PMC8811147; doi:10.3389/fimmu.2022.827865)

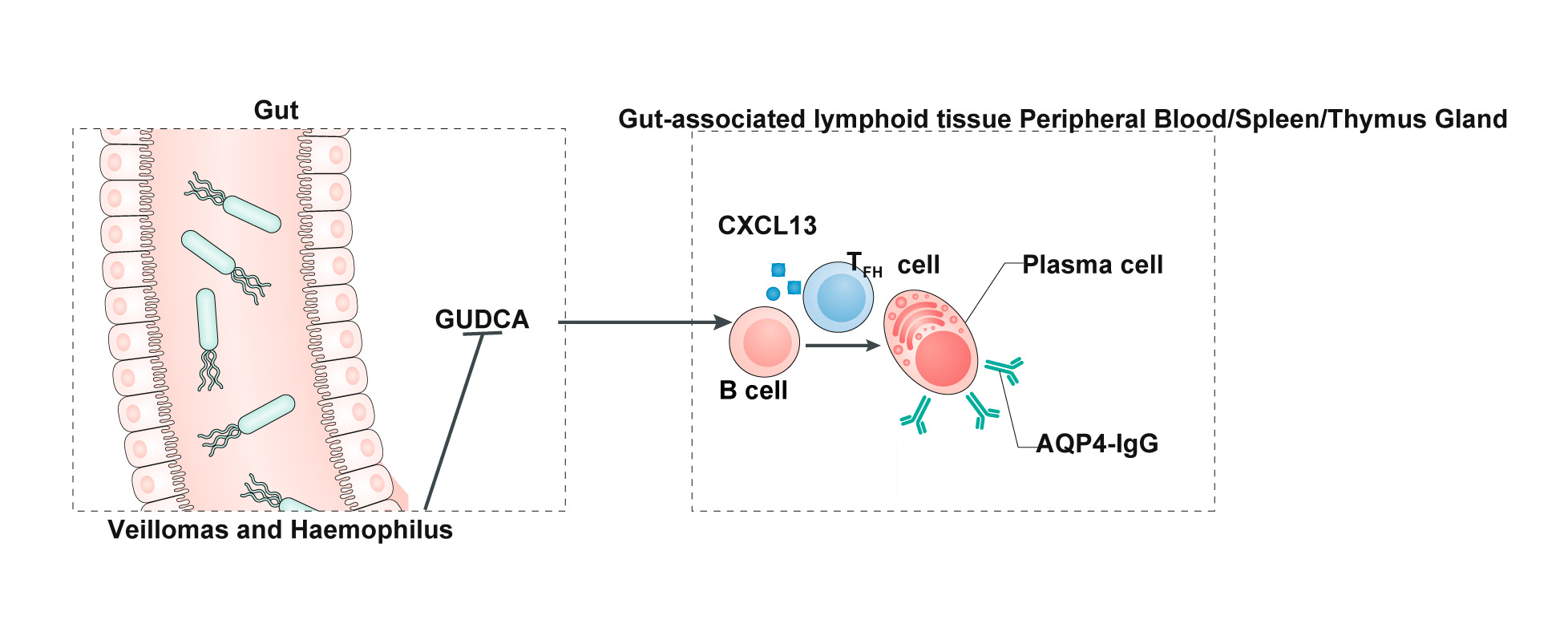

Supplement: Supplementary file 1 [file Image_1.jpeg]
